# Exploring the application of the microbial pigment violacein as a sustainable probe molecule for recycled paper-based SERS substrates

**DOI:** 10.3389/fchem.2025.1571986

**Published:** 2025-05-21

**Authors:** Alessia Tropea, Donatella Spadaro, Dario Giuffrida, Sebastiano Trusso, Daniele Giuffrida, Tania Maria Grazia Salerno, Julio Montanez, Lourdes Morales-Oyervides, Luigi Mondello, Rosina Celeste Ponterio

**Affiliations:** ^1^ Messina Institute of Technology c/o Department of Chemical, Biological, Pharmaceutical and Environmental Sciences, Former Veterinary School, University of Messina, Messina, Italy; ^2^ Institute for Chemical and Physical Processes, National Research Council, Messina, Italy; ^3^ Department of Biomedical, Dental, Morphological and Functional Imaging Sciences, University of Messina, Messina, Italy; ^4^ Facultad de Ciencias Quimicas, Universidad Autonoma de Coahuila, Saltillo, Coahuila, Mexico; ^5^ Chromaleont s.r.l., c/o Department of Chemical, Biological, Pharmaceutical and Environmental Sciences, Former Veterinary School, University of Messina, Messina, Italy

**Keywords:** violacein, recycled-paper, municipal solid waste, SERS sensor, SERS probe, circular bioeconomy

## Abstract

Paper and board represent 10%–39% of the total municipal solid waste generated. In order to address the European Commission (EC) recycling targets, this study aimed to develop flexible Surface-enhanced Raman Scattering (SERS) recycled-paper-based substrates tested by using a new eco-friendly and nontoxic molecule probe. This study reports for the first time the implementation of the microbial dye violacein, obtained by *Janthinobacterium lividum* batch cultivation, as SERS probe, as a suitable substitutive to the most employed toxic chemical dye Rhodamine 6G (R6G). The interaction of the proposed natural probe with the metal surface after the adsorption and the presence of local electromagnetic fields were evaluated by computational approach. The SERS devices developed were decorated by applying a single-step pulsed laser deposition (PLD) decoration method and characterized using UV–Vis absorption spectroscopy. The platform showed a remarkable sensitivity, achieving a detection limit of 10^–7^ M for violacein, allowing to point out the strong potential of this natural microbial dye as a sustainable probe molecule for low-concentration analytes detection on SERS-active substrates, making them suitable for several application fields, such as environmental monitoring, food safety, cultural heritage analysis and diagnostics. This study demonstrates the feasibility of implementing eco-friendly materials in the development of chemical sensors as a sustainable innovation in environmental science by minimizing the ecological impact.

## Graphical Abstract

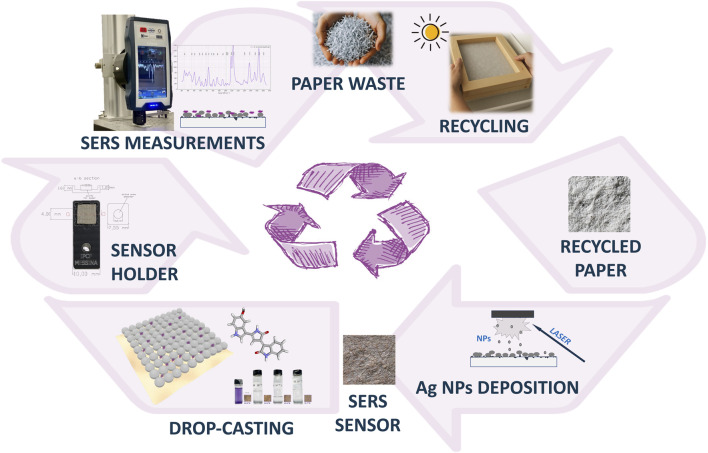

## 1 Introduction

The population growth, expected to reach 4.8 billion inhabitants by 2030, and the consequent increase of urbanization and industrialization are constantly contributing to the generation of municipal solid waste (MSW), whose production is expected to reach 4,2 billion tonnes by 2050 (Serrano et al., 2025; Vinti et al. 2021). MSW are mainly represented by food, gardening wastes, metal, plastics, textiles, glass, electronics, paper, board and miscellaneous waste. Among these, paper and board have been reported to range from 10% to 39% of the total, highlighting that an important amount of paper waste is generated instead of being recycled into new products (Hietala et al., 2018).

The incorrect MSW disposal is generating significant concerns among the World Health Organization (WHO) due to its potential to cause environmental pollution, which is strictly linked to adverse health effects for the population (World Health Organization, 2015).

Moreover, the United Nations (UN) suggested 17 Sustainable Development Goals (SDGs) to guarantee global sustainable development by 2030, emphasizing environmental protection and public health through recycling strategies (United Nations, 2015; Szarka et al., 2021). Concerning MSW, the European Commission (EC) has established specific recycling targets mandating that by 2035, a minimum of 65% of MSW must be recycled. Furthermore, more stringent regulations are imposed on recycling packaging waste, stipulating that 85% of paper and cardboard packaging must be recycled by 2030 (European Commission, 2018).

From this point of view, paper recycling has been explosive in the last decades, both for paper and cardboard new products as well as for new industrial sectors such as the construction materials industry, electronics, agriculture, pharmaceutical, biomedical, food and feed industry and optical sensor (Nechita, 2020; Wang et al., 2021; Javanbakht and Namazi, 2018; Ma et al., 2015). The 3D porous structure, flexibility, lightweight, biodegradability, and affordability make the paper a suitable and interesting substrate for the development of optical sensors (Wang et al., 2021). The interest in this matter is significantly heightened when the substrate is constituted of recycled paper, as recycling represents a fundamental feature of product design from a circular bioeconomy view.

Recently, the cost-effective, environmentally friendly, and renewable properties of paper have garnered significant attention, leading to numerous studies on its implementation as a flexible substrate platform for Surface-enhanced Raman Scattering (SERS) (Liu et al., 2016; Liu et al., 2020; Giuffrida et al., 2025; Park et al., 2018; Ogundare and van Zyl, 2019; Suleimenova et al., 2024). Moreover, paper-based sensors exhibit excellent capillary action, allowing them to absorb sample solutions efficiently, enabling a rapid adhesion of the target molecules and the sensor surface enrichment (Singh et al., 2018; Vo et al., 2019).

SERS technology is a powerful analytical technique showing great applicability in biochemistry, materials research, and environmental monitoring. It provides excellent specificity and sensitivity for the detection and the characterization of molecular species at the nanoscale by utilizing the Raman scattering and plasmonics principles (Zangana et al., 2024). SERS can be described as a surface phenomenon that allow the Raman scattering signal amplification of molecules adsorbed on a plasmonic metal nanoparticle when it is excited with a proper laser wavelength. This amplification is responsible of the enhanced sensitivity and the improved signal-to-noise ratio (Bodelón et al., 2018).

In recent year, this technique is gaining increasing attraction in several research fields, such as trace molecular detection (Fierro-Mercado and Hernández-Rivera, 2012; Moram et al., 2018; Sijia et al., 2020; Zangana et al., 2024), clinical diagnostics and therapeutics (Khan et al., 2022; Usman et al., 2023; Gao et al., 2023; Benešová et al., 2023), bacteria identification (Wang and Luo, 2023; Bodelón et al., 2017), food safety sensing (Xiong et al., 2018; Xiong et al., 2017), because of its several advantages in comparison with other analytical methodologies, such as fingerprint recognition, real-time applications, minor or no sample preparation, no water interferences, high resolution, and non-destructiveness of the sample (Fierro-Mercado and Hernández-Rivera, 2012; Usman et al., 2023; Suleimenova et al., 2024).

The implementation of flexible SERS substrates is emerging as a new innovative sensing platform generation, especially if compared with conventional rigid SERS substrates, because of their interesting application for analytes monitoring from irregularly shaped surface and on 3D objects. Moreover, the flexible feature offers more “hot spots” determining stronger SERS enhancement (Sijia et al., 2020; Wang and Luo, 2023). In order to verify the sensitivity and reproducibility of new SERS substrates, the most employed Raman probe is represented by Rhodamine 6G (R6G) (Hwang and Yang, 2018; Zeng et al., 2019; Wu et al., 2015; Araújo et al., 2017; Kim et al., 2017; Wang and Luo, 2023; Zangana et al., 2024). R6G has been classified as a toxic chemical dye, able to cause serious damage both to the human health and to the ecosystem, even at concentrations lower than 1 mg/L (Gear, 1974; Nestmann et al., 1979; John, 1989; Phuong et al., 2022; Thaler et al., 2008), determining the need to address the research towards the implementation of new eco-friendly and non-toxic pigments as suitable probes.

With this aim, previous studies have been carried out by using synthetic food dyes, including sunset yellow (SY), and tartrazine (TZ), as standard probe molecules (Barveen et al., 2021; Xie et al. 2014). A significant drawback associated with the implementation of these molecules is the growing concern over the potential hazardous effects that synthetic molecules may pose to both the environment and human health (Rollini et al., 2022; Li et al., 2022).

Currently the 80%–90% of dyes are produced by chemical synthesis. The main drawback associated with the implementation of chemical synthesis procedures is the generation of toxic waste that can harmfully affect both the environment and the human health (Joshi et al., 2023). This aimed the scientific interest on researching for suitable alternatives such as the implementation of natural pigments obtained by vegetable biomasses extractions (Yadav et al., 2023; Yusuf et al., 2017). On the other hand, vegetable-derived dyes are costly because of the long cultivation times required, the plant dependence to climatic conditions, and the extensive land and water resources needed (Lopez et al., 2023).

Moreover, the large-scale production of vegetable biomass for pigments extraction is generating concerns for the ecosystem due to deforestation and infringement on the diversity of local species. Thus, the prevailing challenge for both scientists and industries is to meet the increasing consumer demands for safer and “*natural”* alternatives.

Microbial dyes can represent an excellent alternative, since they present notable advantages over plant-based dyes. These include the rapid growth of microorganisms, their independence from seasonal limitations, and the possibility of their cultivation under strictly controlled conditions in bioreactors. Additionally, microorganisms can produce a diverse range of pigments according to the species and cultivation conditions employed (Narsing Rao et al., 2017; Rajendran et al., 2023). Advances in biotechnology development further enhance the potential for eco-sustainable processes to be applied across various sectors.

To the best of our knowledge, the implementation of natural dyes, particularly microbial dyes, as SERS probes, has not been previously explored. In this study, we report the implementation of paper waste as a flexible substrate for SERS platform, evaluating the sensors performance with violacein ([3-(1,2-dihydro-5-(5-hydroxy-1H-indol-3-yl)-2-oxo-3H-pyrrol-3-ilydene)-1,3-dihydro-2H-indol-2-one]), an indole derivative compound with a deep purple hue, produced via batch cultivation of the bacterium *Janthinobacterium lividum*. Violacein has been largely studied for its antimicrobial, antileishmanial, antiviral, as well as antitumor properties, and it has also been used as a colorant for a variety of natural and synthetic fabrics instead of other chemical colorants for textile dyeing (Kanelli et al., 2018; Durán et al., 2007; Rettori and Durán, 1998. However, no studies have reported its application as a probe molecule.

Moreover, a single-step decoration method, previously described (Giuffrida et al., 2025), based on Pulsed Laser Deposition (PLD) (Fazio et al., 2013; Agarwal et al., 2014), was applied in order to further reduce the environmental negative impact due to chemicals or thermal treatment. Finally, Density Functional Theory (DFT) calculations were performed to obtain the vibrational properties of violacein and deoxyviolacein molecules to be compared to experimental spectra (Jehlička et al., 2015).

The research was addressed on an ecological platform development aligned with the cradle-to-cradle concept. The SERS platforms utilized in this study can be recycled and converted into new, functional substrates, eliminating the need for introducing synthetic and/or toxic molecules into the environment.

## 2 Materials and methods

### 2.1 Materials and chemicals used

Recycled cellulose substrates from wastepaper were produced by an artisanal and sustainable process to eliminate unwanted chemicals in the final product. Paper drying was performed without the use of artificial heat sources (*Angolo del CARTigianato* -Reggio Calabria Italy). The paper showed a rough textured surface, interlaced with a complex web of fibers, providing a distinctly organic feel. The colour was a homogenous light grey, punctuated by tiny flecks and specks of darker material as a result of the recycled origin of the paper. The edges were somewhat frayed due to the handcrafted quality.

Ag nanoparticles produced by PLD starting from silver target 99.99%, dia. 15 mm, thick 1 mm from Nanovision srl.

Peptone, glucose, sodium chloride (NaCl), Dipotassium phosphate (K_2_HPO_4_), and agar were provided by Merk (Merck Lifescience, Damstad, Germany).

Ethanol (hypergrade LC-MS) was used to dissolve the samples. Water, acetonitrile (both MS grade) and formic acid were used for HPLC-DAD-ESI-MS analyses. All the solvents were obtained from Merck (Merck Lifescience, Damstad, Germany).

### 2.2 Microorganism and inoculum conditions

The strain employed in this study was *Janthinobacterium lividum* CECT946. It was maintained in a cryoprotective medium consisting of skim milk-glycerol (10% skim milk, 10% glycerol) stored at −20°C. Initially, the strain was reactivated by streaking onto Petri dishes. The growth medium comprised the following (g/L): peptone (17), glucose (2.5), NaCl (5), K_2_HPO_4_ (2.5), and agar (20). The pH of the medium was adjusted to 7.2 and sterilized in an autoclave (Sterilmatic STME, United States) at 121°C and 15 psi for 15 min. Incubation of the strain was conducted at 21°C for 56 h. Subsequently, the strain was transferred to shake flasks (125 mL) containing the aforementioned medium (25 mL, without agar).

### 2.3 Violacein production and recovery

The production of violacein was executed at a shake flask level utilizing the previously described medium, but with molasses and sodium glutamate as carbon and nitrogen sources, respectively. The medium composition was as follows (g/L): molasses (2.5), sodium glutamate (23.0), NaCl (5.0), and K_2_HPO_4_ (2.5). Fermentation media was adjusted to pH of 7.2. The production process was carried out at 21°C and 150 rpm for 63 h, with an inoculum size of 10% (v/v) in an orbital incubator (Innova 94, New Brunswick Scientific, United States). To recover violacein, the fermented broth (20 mL) underwent centrifugation at 6,235 g and 4°C for 20 min, separating the supernatant from the cellular pellet. The pellet was washed with sterile distilled water and frozen until further extraction process (Wang et al., 2009). To recover the pigment, it was utilized a horn-type ultrasound (Sonic Vibracell, United States) with ethanol as the solvent (working volume 20 mL). The extraction conditions included an amplitude of 40%, an extraction time of 4 min, and a solid-to-liquid ratio of 1:10. Following the extraction, the biomass was separated by centrifugation at 6,236 g for 20 min at 4°C. Subsequently, ethanol was removed using rotary evaporation. The pigmented extract was then subjected to freezing at −20°C and subsequent lyophilization, resulting in the production of a violet-colored powder.

### 2.4 HPLC-MS-PDA analysis

The lyophilized powder obtained from *Janthinobacterium lividum* was dissolved in 1 mL of ethanol and subsequently filtered through a syringe filter (PTFE, 0.45 μm). The ethanolic solution was stored in fridge at 4°C until HPLC analyses were carried out. A Shimadzu Prominence UFLC XR system equipped with a CBM-20A Controller, a SIL-20A XR Autosampler, two LC-20AD XR Solvent Delivery Unit and a CTO-20A Column Oven was employed for HPLC analysis. The instrument was coupled to an SPD-M20A Photo Diode Array Detector and to an LCMS-Mass Spectrometer detector (Shimadzu, Duisburg, Germany).

Chromatographic separation was achieved an Ascentis® Express C18 column (100 × 2.1 mm; 2.7 μm, Merck KGaA, Damstad, Germany) employing water and acetonitrile (both acidified with 1% of formic acid) as eluent A and B, respectively. Gradient went from 40% to 80% of B in 10 min, flow rate was of 0.2 mL/min, sample injection volume was 1 μL, and an oven temperature was set to 30°C. DAD detector was acquiring in the 190–800 nm range and spectra were visualized at λ = 570 nm. Mass spectra were acquired employing an electrospray interface (ESI) operating in both positive and negative ion modes. Mass spectra were acquired in the range 180–700 *m/z* with a scan speed of 1875 u/sec. DL and heat block temperature were set at 250°C and 400°C, respectively. Nebulizing gas flow was 1.5 L/min and Drying gas flow was 10 L/min.

LabSolutions software ver. 5.82 (Shimadzu) was used for analysis and data processing. Identification of compounds was accomplished using both DAD and MS data acquired.

### 2.5 Extract optical properties investigation

The optical properties of the violacein solution have been investigated using Ultraviolet-Visible Spectroscopy (UV/Vis). Analyses were carried out by using a 190–1,100 nm scan of the pigment solutions by using a V-730 spectrophotometer (Jasco). The solvent used for the pigment resuspension, ethanol, was used as blank.

### 2.6 Fabrication of the SERS devices based on recycled cellulose substrates and Ag nanoparticles obtained by pulsed laser deposition

SERS substrates were produced in the laboratory from recycled paper decorated with Ag nanoparticles obtained via Pulsed Laser Deposition (PLD) (D’Andrea et al., 2009; Ossi et al., 2011; Fazio et al., 2009). The well-established deposition protocol, reported in a previous study, allows for covering the cellulose surface with a nanostructured thin film, where couples of Ag nanoparticles with dimensions of about 20 nm, generate localized hotspots which enhance the signals of adsorbed molecules (Fazio et al., 2014; Mollica Nardo et al., 2018).

In this process, the nanoparticle (NP) size is influenced by a delicate interplay of several factors, such as laser fluence, distance between the target and substrate, gas type and pressure inside the vacuum chamber, amount of material ablated per pulse (Zhan et al., 2018; Hutter and Fendler, 2004; Willets and Van Duyne, 2007. These factors have a significant impact on the film’s properties and consequently affect SERS activity. The most effective SERS performance occurs when the excitation wavelength is matched to the localized surface plasmon resonance of the nanostructures.

**FIGURE 1:**
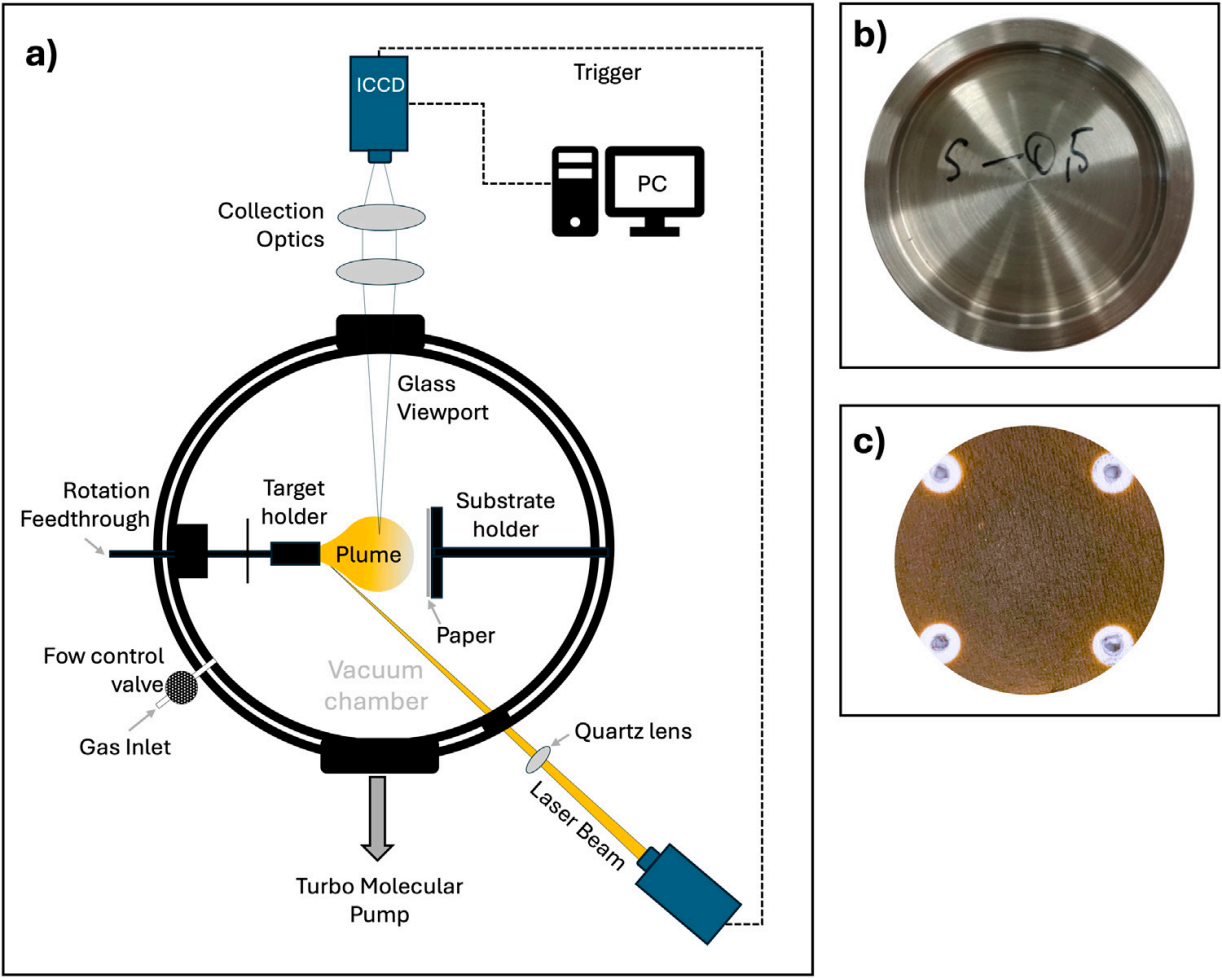
Scheme of Pulsed Laser Deposition process **(a)**; aluminum paper substrate holder **(b)**; Ag deposited paper **(c)**.

In our previous study (Giuffrida et al., 2025), we optimized the deposition protocol to suit the cellulose substrate, by tuning the sample holder within the deposition chamber and the environmental conditions (Figure 1). Paper-based samples are, indeed, highly flexible and fragile, making it impractical to use the same sample holder designed for glass and silicon fragments. To address this challenge, a custom sample holder was specifically designed and manufactured to securely accommodate cellulose substrates within the deposition chamber (Figures 1a–c). This included a set of aluminum masks to lock the paper in place on the support, ensuring stability during the deposition process. The morphology of plasmonic nanostructures has been widely investigated in a preliminary study (Giuffrida et al., 2025), in relation not only to the deposition process but also to the type of paper used, in particular for texture and roughness to choose the best for SERS application.

### 2.7 Preparation violacein solution

**FIGURE 2:**
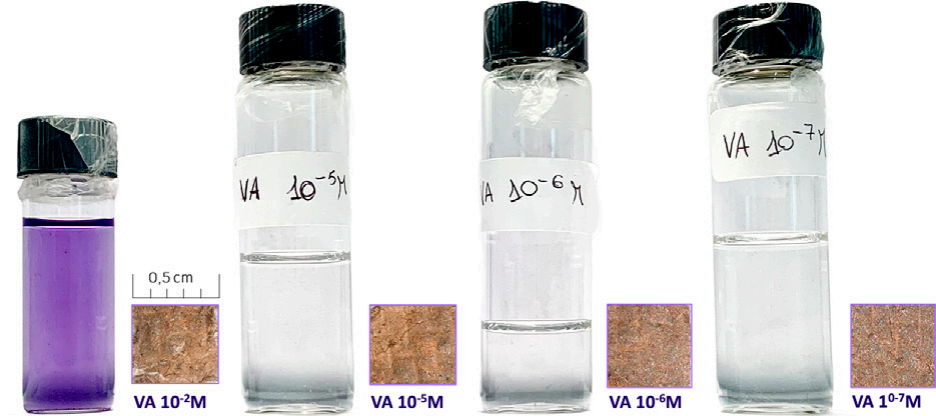
Violacein solution at progressive concentration and SERS platforms drop-casted with violacein at different concentrations from 10^−2^ M to 10^−7^ M.

To explore the effectiveness of violacein in SERS applications, solutions with varying molar concentrations (10^−2^ M, 10^−5^ M, 10^–6^, 10^−7^ M) of violacein were prepared and drop-casted on the SERS platforms, previously tested and optimized which lack evident color even at the highest concentrations (Figure 2). Tests were conducted on paper type, which had demonstrated the best performance using Rhodamine 6G solution as the probe molecule with a lower detection limit around 10^–10^ (Giuffrida et al., 2025).

### 2.8 Raman/SERS spectroscopy

**FIGURE 3:**
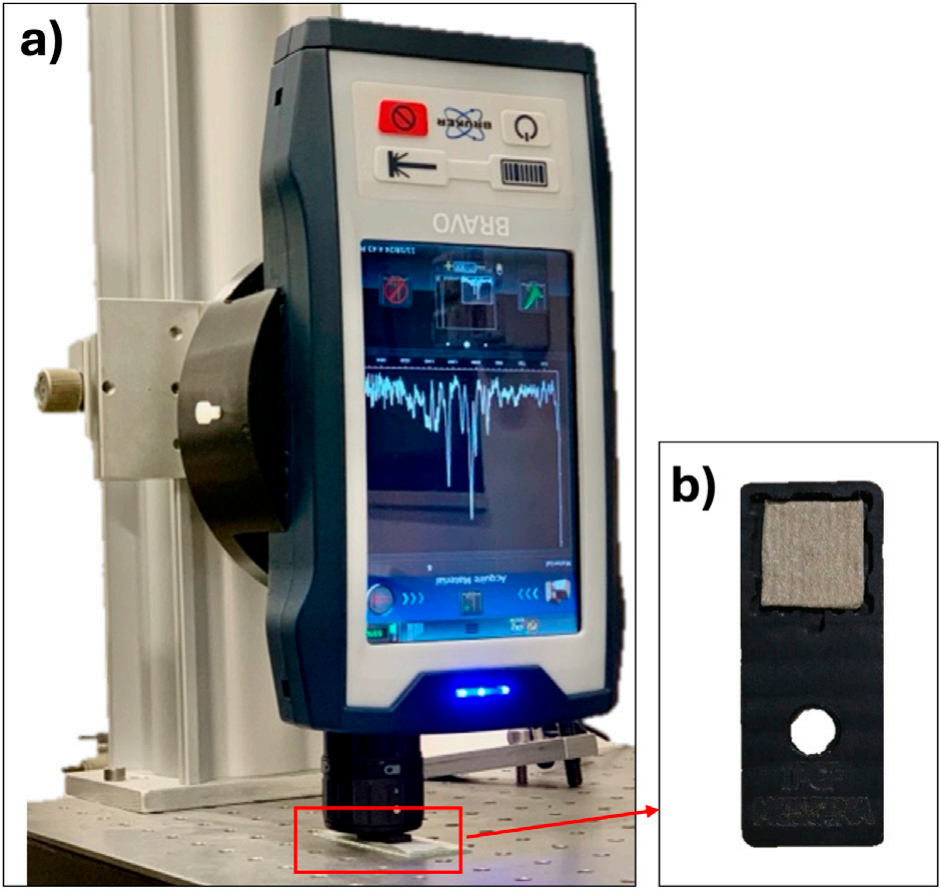
Bravo-Bruker spectrometer during acquisitions **(a)** on the SERS platform; **(b)** prototype of sensor holder for SERS applications.

Raman spectroscopy is a highly efficient technique that enables the identification of various materials. This method relies on the inelastic scattering of light by molecules or materials, providing insights into their vibrational modes and structural and electronic properties (Eesley, 2013). It is fast, non-destructive, and offers high sensitivity for recognizing different species. Additionally, it can be integrated with advanced optical microscopy, optical fibres, miniaturized lasers, and other photonic devices to enhance diagnostic capabilities (Ferrari and Basko, 2013). For example, metal nanoparticles are used to significantly amplify Raman signals through their plasmonic response. Surface-enhanced Raman scattering (SERS) spectroscopy is based on the phenomenon where Raman signals from molecules adsorbed on rough surfaces or nanoparticles made of noble metals (typically gold and silver) are greatly enhanced when illuminated by a laser. This enhancement arises from two effects: a weak chemical mechanism and a stronger electromagnetic mechanism, both linked to the resonant interaction of light with localized surface plasmons (LSPs) excited in the nanoparticles. The aggregation of metal nanoparticles creates “hot spots,” which are nanocavity regions between adjacent particles that further amplify the Raman signal of adsorbed molecules (Fan et al., 2011; Camposeo et al., 2016). In this study, Raman spectroscopy was employed to evaluate the SERS activity of these violacein solutions drop-casted on the SERS substrates and dried naturally. Raman spectra were collected using the BRAVO™ (Bruker Optik GMBH, Germany) handheld Raman spectrometer. This instrumentation can exhibit improved performance in acquiring SERS spectral signals under specific sample types and testing conditions thanks to the employed technology, which optimizes signal acquisition depending on the sample’s characteristics (Figure 3).

The BRAVO utilizes a dual-laser system centered at 785 nm and 853 nm (DuoLaser™), with each laser covering a different spectral region and partially overlapping, resulting in spectra recorded across the 300 to 3,200 cm^−1^ range. The spectral resolution ranges between 10 and 12 cm^−1^. The device incorporates patented SSE™ (Sequentially Shifted Excitation) technology for fluorescence suppression. This technology operates the laser diodes at different temperatures, causing minor shifts (<1 nm) in excitation wavelengths. Non-Raman signals, such as spectral artifacts and fluorescence bands, remain at fixed positions within the spectral space, while Raman bands shift slightly based on the changes in excitation wavelengths. This allows for computational subtraction of the fluorescence signal, obtaining a “clean” final Raman spectrum.

During acquisition, the instrument was connected to a computer via Wi-Fi for manual acquisition parameter control through OPUS software. Raman spectra were collected from multiple points on each substrate to ensure the uniformity of the signal using 1,200 ms × 3 scans as acquisition parameters.

The SERS measurements on the platform were conducted using a prototype substrate holder specifically designed and manufactured to align with the Bravo Bruker, as well as other Raman spectrometers. In this configuration, acquisitions are made by orienting the substrate holder in a horizontal position on an optical bench, while aligning the Raman instrumentation in a vertical orientation (Figure 3).

## 3 Results and discussion

### 3.1 Pigment characterization through HPLC-MS-PDA and spectrophotometric analyses

**FIGURE 4:**
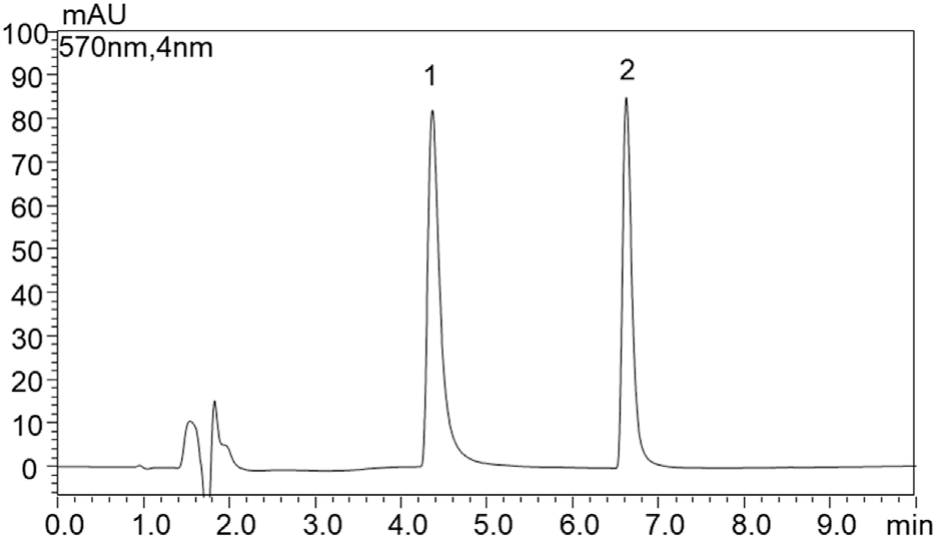
HPLC-PDA chromatogram analysis of the pigment extract from *Janthinobacterium lividum* (λ = 570 nm) where AU is for absorbance unit.

**FIGURE 5:**
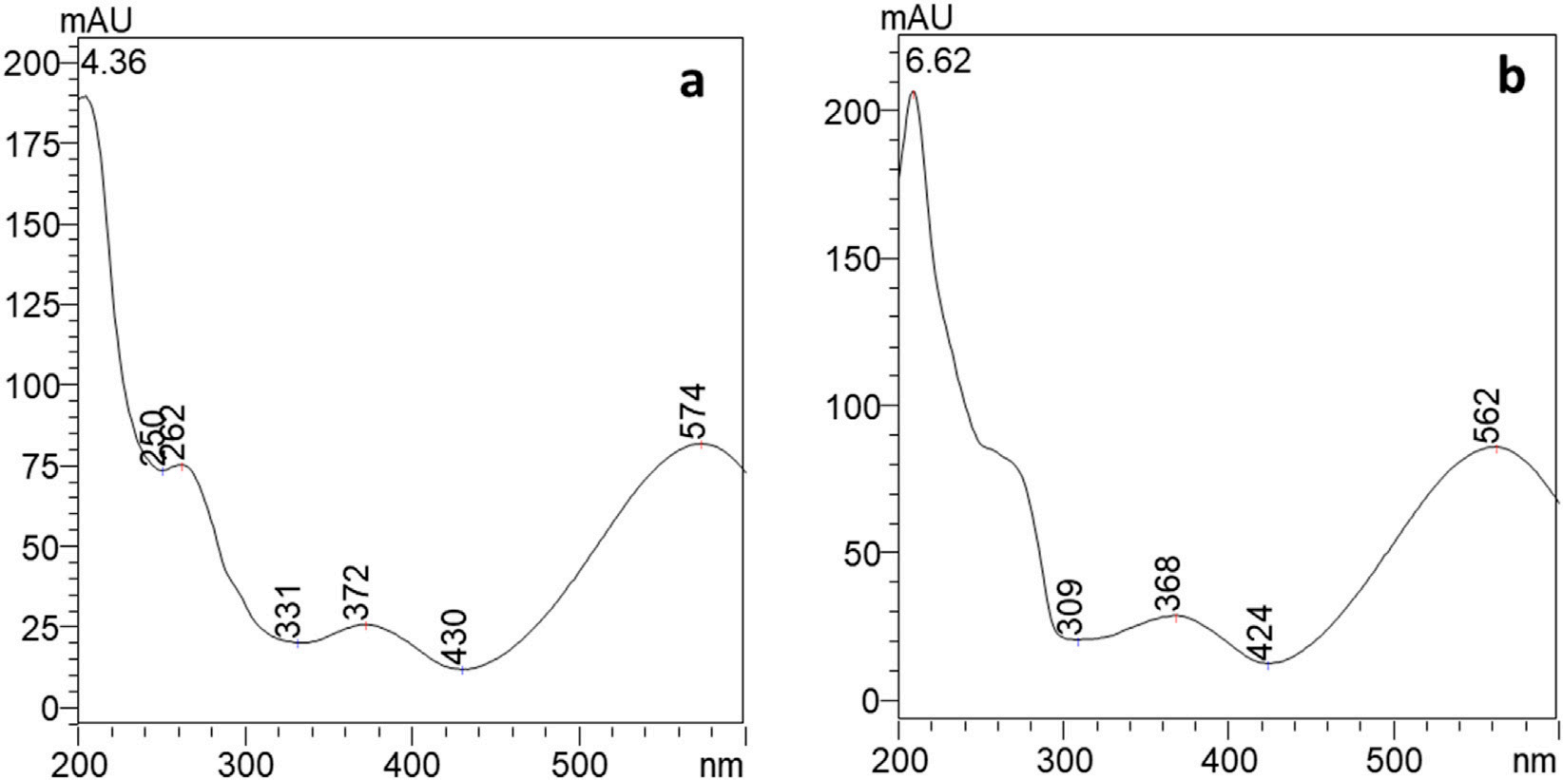
UV/Vis spectra of compound eluting a 4.4 min, peak 1 **(a)** and at 6.6 min, peak 2 **(b)** where AU is for absorbance unit.

Extracts dissolved in ethanol were subject to HPLC-PDA-ESI-MS analysis following the condition detailed in the materials and methods section. Two compounds, eluting at 4.4 min and 6.6 min, have been detected (Figure 4). The two compounds showed similar absorption spectra with a maximum at λ = 574 nm for peak 1 and at λ = 562 nm, slightly blue shifted, for peak 2 (Figure 5). The HPLC results showed that the pigments produced by *Janthinobacterium lividum* contained 57.36% of compound 1% and 42.64% of compound 2.

**FIGURE 6:**
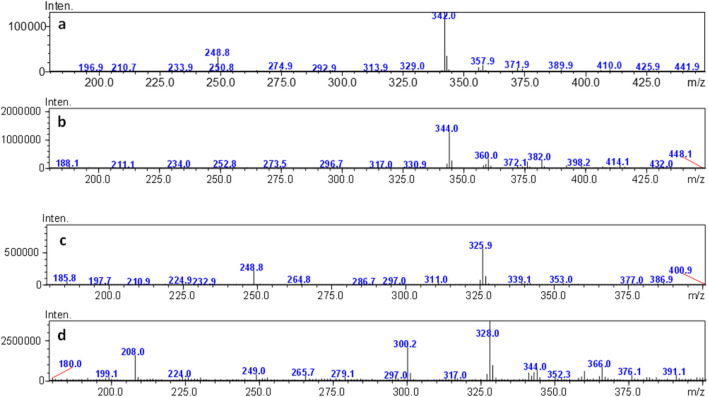
ESI-MS spectra of compound eluting a 4.4 min, obtained in negative **(a)** and positive ionization mode **(b)**; ESI-MS spectra of compound eluting a 6.6 min, obtained in negative **(c)** and positive **(d)** ionization mode.

Compounds were identified using mass spectrometry employing an electrospray interface. In detail, for the first eluted compound (peak 1 in Figure 4), a molecular ion [M-H]^−^ was observed at *m/z* 342.0 for mass spectra acquired in negative ionization mode. Conversely, 344.0 *m/z* was the molecular ion **[M–H]^+^** recorded in positive ionization mode. These masses are consistent with those of violacein (C_20_H_13_N_3_O_3_). For the compound eluting at 6.6 min (peak 2 in Figure 4), [M-H]- was found at 325.9 *m/z* and **[M–H]^+^** at 328.0 *m/z* (Figure 6). Hence, the second peak can be attributed to deoxyviolacein (C_20_H_**13**_N_3_O_**2**_) (Wang et al., 2012). These results are consistent with previous ones where violeacein and deoxyviolacein have been identified as the main pigments produced by *J. lividum (* Choi et al., 2015 *)*.

**FIGURE 7:**
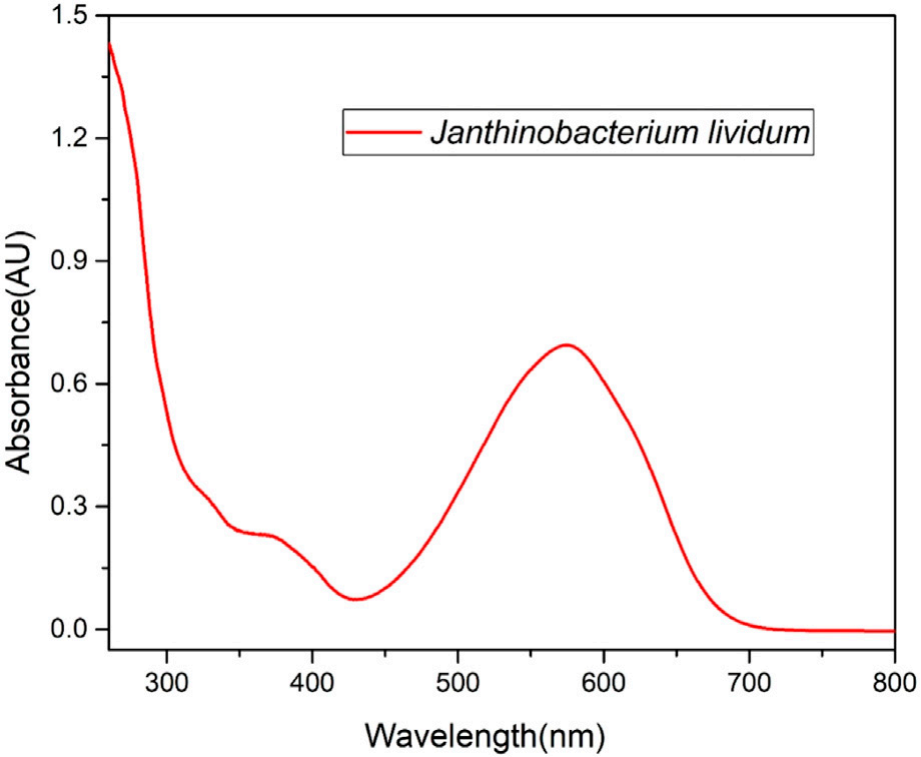
Absorbance spectrum of a solution of Jantinobacterium lividum extract in ethanol.

A UV-Vis spectrophotometric measurement was performed on the solution prepared for SERS testing to confirm its physicochemical characteristics. The absorption spectrum in Figure 7 displays two absorption bands maxima at 370 and 575 nm and it was confirmed to be similar to the violacein standard spectrum (Anahas et al., 2022; Dantas et al., 2013) and to the compounds eluted from HPLC analyses.

### 3.2 Substrate characterization

**FIGURE 8:**
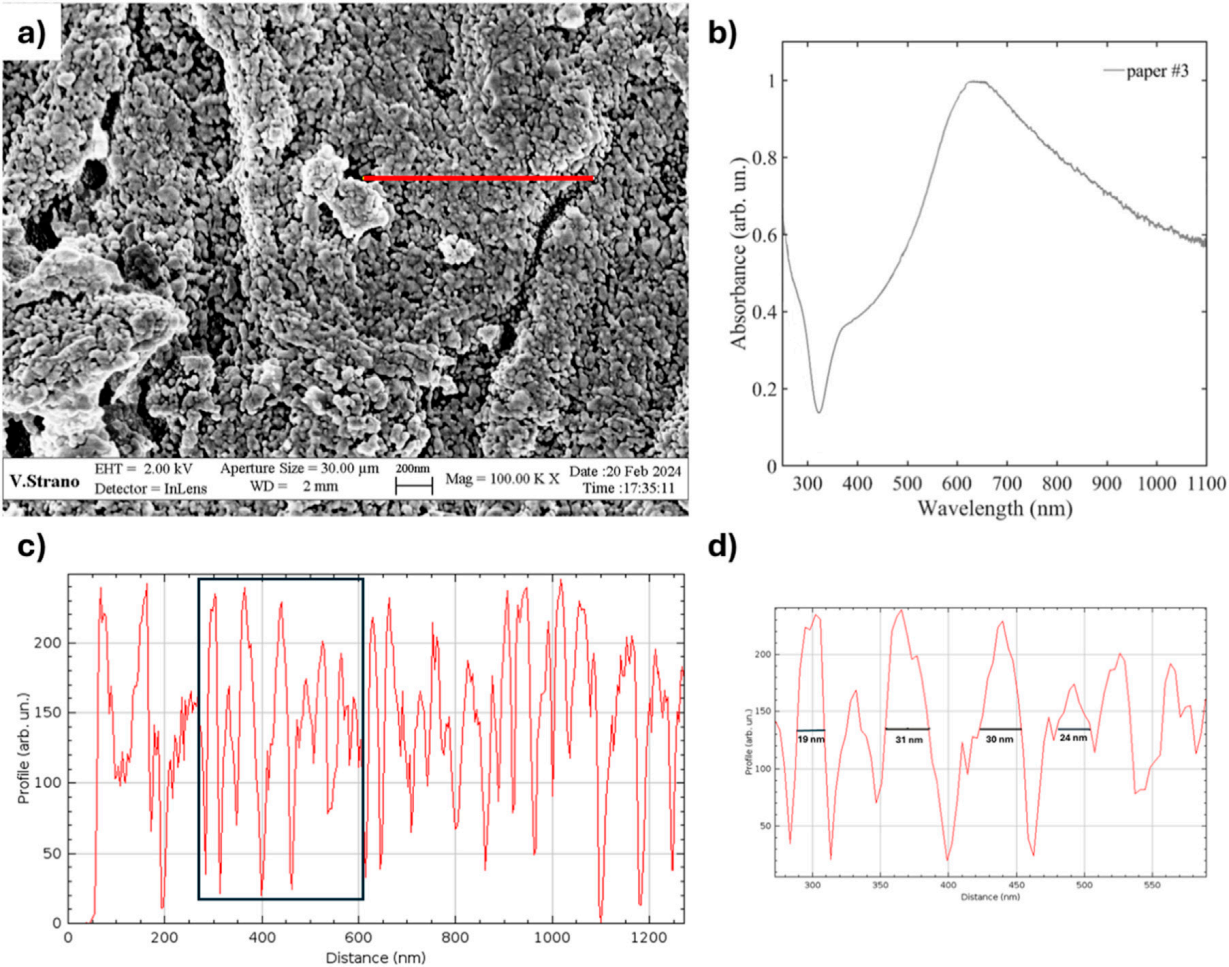
**(a)** SEM image showing the distribution of the Ag NPs onto the cellulose fibers composing the paper substrate (the red line represents the region investuigated for NPs size estimation); **(b)** UV–Vis spectrum acquired on glass substrates coated with Ag NPs using the same deposition processes employed for paper #3 substrate; **(c)** Profile extracted illustrating surface variation; **(d)** Magnified view of a selection of the profile, highlighting nanostructure spacing.

The cellulose substrate before deposition was characterized via laser profilometry and results are reported in Giuffrida et al., 2025 (paper #3). Scanning Electron Microscopy (SEM) imaging was used to investigate the Ag nanoparticle distribution deposited by PLD onto the paper surface and assess their homogeneity, while UV-VIS absorption spectroscopy measurements were performed on the glass slide positioned beside the paper sheets during the deposition to track the position of the surface plasmon resonance peak (Figure 8). In Figure 8a, the red line represents the region analyzed for nanoparticle size estimation. The UV–Vis spectrum (Figure 8b) is characterized by the presence of a broad absorption band around 750 nm and extending between 350 and 1,100 nm.

Figure 8c provides an estimation of the NPs distribution and size along the selected profile line (for comparison, see also Giuffrida et al., 2025 and Romo-Herrera et al., 2021). While localized variations are present, the overall nanostructured morphology is consistent across the analyzed region. The images were processed and scaled in ImageJ using the embedded scale bar to ensure accuracy. The observed variations in the intensity profile do not correspond to the real elevation but rather to the distribution of Ag nanoparticles on the surface. The nanoparticle dimensions (Figure 8c, d) and distribution calculated in this study are fully consistent with with that obtained from other SEM images reported in our previous article, showing a size range approximately of 19–31 nm. This agreement is expected, as the deposition was carried out simultaneously and under identical experimental conditions. Further statistical analysis across multiple areas confirms the homogeneity of the pattern, ensuring substrate reproducibility.

### 3.3 Violacein tested as the probe molecule on the SERS device

**FIGURE 9:**
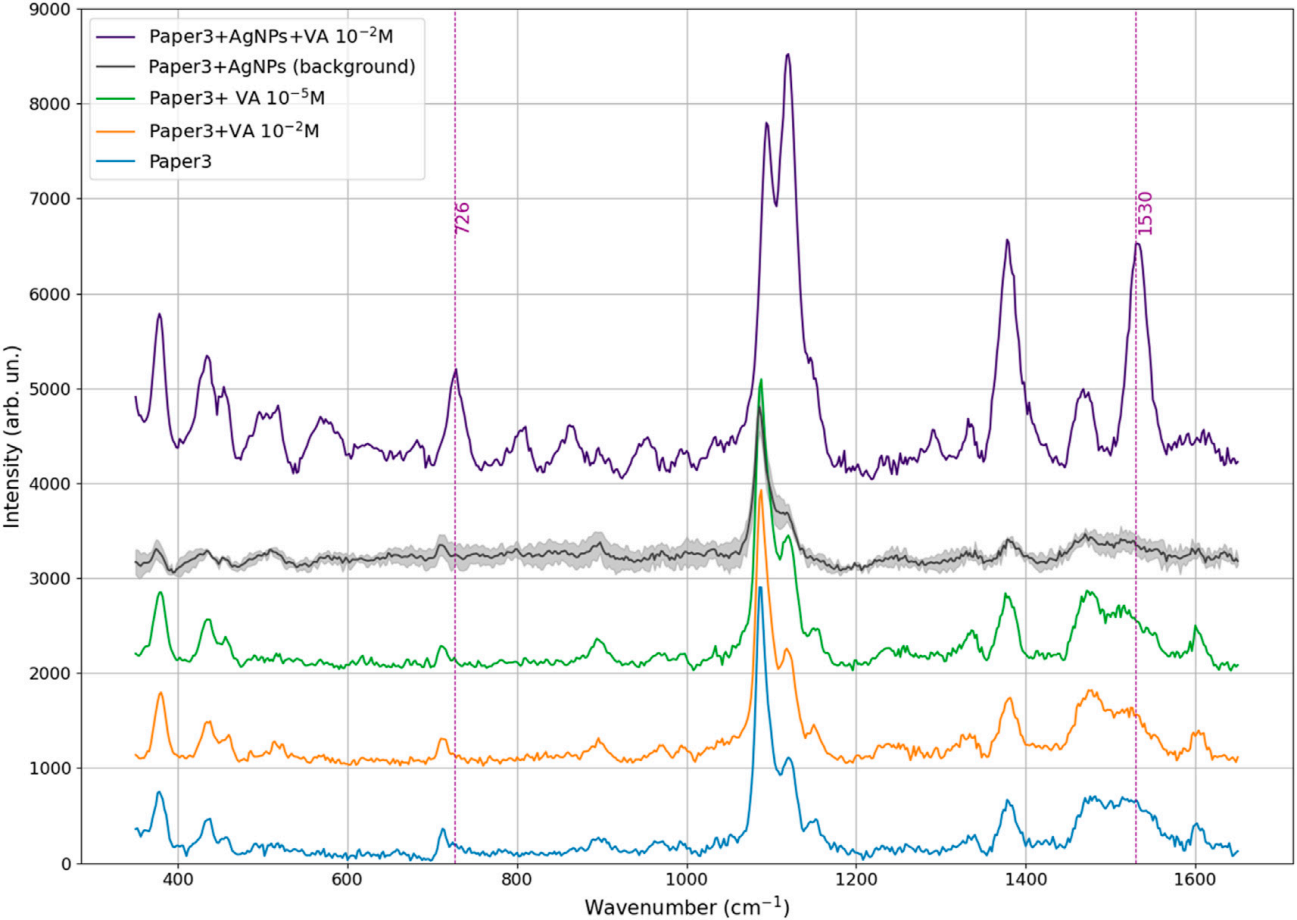
Raman spectra comparing paper substrates and prepared samples (from bottom to top): Paper substrate (blue); Paper with violacein at 10^–2^ M concentration (orange); Paper with violacein at 10^–5^ M (green); Paper covered with Ag nanoparticles (SERS platform), shown as the mean spectrum with standard deviation shading (gray); SERS platform with violacein at 10^–2^ M (purple). Dashed vertical lines indicate characteristic peaks of violacein at 726 and 1530 cm^–1^.

As the first step, spectra were acquired from the recycled paper substrate alone (Figure 9), the same substrate with violacein at 10^–2^ M Ag Nps, and SERS substrate with Ag nanoparticles without violacein. As shown in Figure 9, the plain recycled paper exhibits the characteristic Raman peaks of cellulose, while no violacein-specific bands (e.g., 726 and 1,530 cm^–1^) are distinguishable on the paper substrate without Ag NPs even at the highest concentration tested (cfr. Agarwal et al., 2021). The bare SERS substrate (paper + AgNPs) shows a general reduction in cellulose peak intensity due to plasmonic damping effects.

As the second step, SERS measurements were conducted with violacein (10^–2^ M) on Ag-deposited platforms. The comparation between violacein at 10^−2^ M drop-casted on plain cellulose and Ag nanoparticle-coated substrates (Figure 9), respectively, revealed a marked enhancement in Raman signal upon nanoparticle decoration. While the spectrum collected on the plain paper (without deposition) shows only the peaks related to cellulose structure, given the low concentration of the probe molecule, vibrational modes attributed to violacein’s molecular structure, mainly those centered at 728 cm^–1^, 946 cm^–1^, and 1,530 cm^–1^, were amplified on the nanostructured surface by localized surface plasmon resonance effects.

To evaluate the capabilities of the paper-based SERS substrate, a series of Raman ans SERS spectra were collected for the recycled paper and the bare SERS substrate (paper + AgNP), both in absence and presence of the target molecule. Representative spectra are presented below, followed by the corresponding calibration curve and calculation of the enhancement factor (EF) to quantitatively assess SERS performance.

Figure 9 shows the Raman spectra of paper substrates dropcasted with violacein (VA), both with and without silver nanoparticles (AgNPs) and the Ag-deposited. From the Raman spectra is evident that violacein is not detectable on plain paper substrates, even at the highest tested concentration of 10^–2^ M. The spectrum of “Paper3 + VA 10^–2^ M” shows no distinct Raman peaks attributable to violacein emerging from the baseline signal of the cellulose-based substrate (Agarwal et al., 2021). This confirms that the cellulose-based substrate alone is not sufficient to reveal vibrational features of violacein at this concentration. The “Paper3 + AgNPs” spectrum shows suppression of the characteristic cellulose bands, suggestin that the AgNPs interact with the cellulose surface, altering its optical or scattering properties.

In contrast, the spectral profile changes under SERS condition (“Paper3 + AgNPs + VA 10^–2^ M”), where clear and intense vibrational modes characteristic of the target molecule (such as those centered at 728 cm^–1^, 946 cm^–1^, and 1,530 cm^–1^) start to emerge. These results demonstrate that the Ag nanostructured surface acts as an efficient SERS platform, amplifying the adsorbed molecules thanks to the localized surface plasmon resonance effects.

The Ag nanoparticles actes as hotspots, inducing strong electromagnetic fields near the molecule-nanoparticle interface that amplified violacein’s Raman signals by several orders of magnitude, while suppressing the background signals arising from from the cellulose support.

This amplification permits the clear visibility of violacein’s vibrational bands even at concentration that would be otherwise weak or undetectable in standard Raman spectroscopy. Notably, the peaks centered at 728 and 1,530 cm^–1^ demonstrated the highest intensity, making it a reliable indicator for quantifying violacein concentration across the tested range. The peak around 1,380 was excluded because it slightly emerge from the bare SERS substrate.

In Table 1 we reported the assignment of the specific vibrational modes of violacein, as suggested by previous studies (Jehlička et al., 2015) (Table 1).

**TABLE 1:** Violacein extracted from *Janthinobacterium lividum*-Raman shifts of major bands and assignments.

Raman shift (cm^-1^)	Intensity	Assignment
1,530	sh	Pyrrole ring C=C and C-N
1,465	m	Pyrrole ring C=C and C-N
1,379	m, br	Quadrant CNC stretching
1,175	m	C-C stretching
1,148	m	C-C stretching
1,141	s	C-C stretching
1,089	w	—
1,026	vw	—
958	m	—
945	m	C-H bending or ring deformation vibrations
870	m	CN stretching
808	w	—
726	s	Aromatic ring C-C and CCC bending
680	m	Aromatic ring C-C and CCC bending
620	w	Aromatic ring C-C and CCC bending
499	m	CCO and CCN rocking and bending modes
456	m	CCO and CCN rocking and bending modes

**FIGURE 10:**
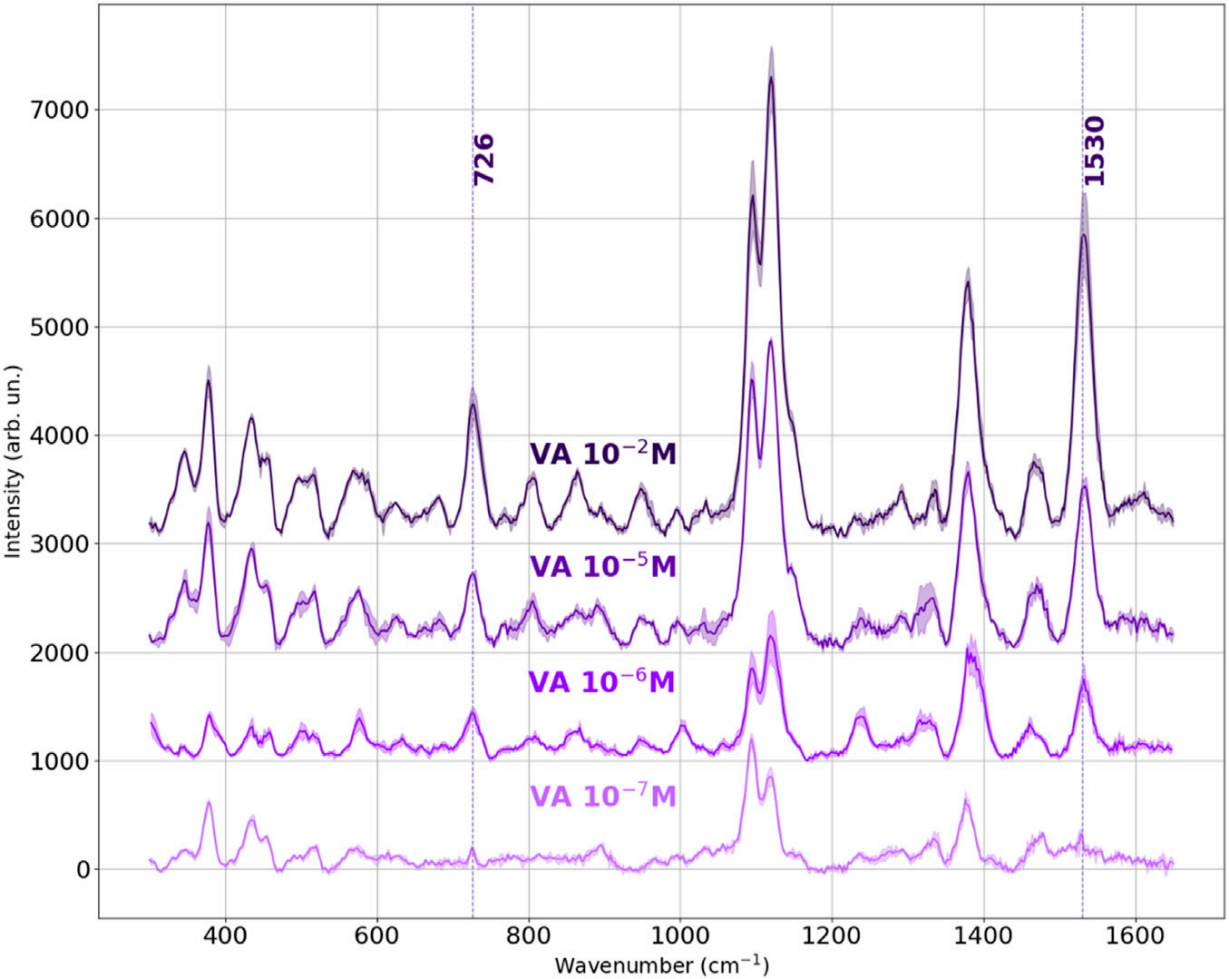
Mean Raman spectra of Violacein at different concentrations (from 10^–2^ M to 10^–7^ M), acquired on the on Ag-deposited SERS platform. For each concentration, the plotted spectrum represents the average of three measurements taken under identical conditions. The shaded regions indicate the standard deviation. The dashed vertical lines mark key peaks at 728 cm^−1^ and 1,530 cm^−1^.

SERS spectra of violacein solutions were collected on the Ag nanostructured platforms across the following concentration rang: 10^−2^ M, 10^−5^ M, 10^−6^ M, 10^−7^ M. In Figure 10, the mean SERS spectra for each concentration are presented, each obtained by averaging three independent measurements under identical conditions. The shaded regions indicate the standard deviation, reflecting the good reproducibility of the measurements. The dashed vertical lines highlight key vibrational bands of violacein, particularly the most intense peaks at 728 cm^−1^ and 1,530 cm^−1^, which were selected as diagnostic markers. A progressive decrease in intensity with decreasing concentration is clearly observed for these peaks, confirming the SERS substrate’s ability to detect violacein down to 10^–7^ M. The scalability of signal intensity with concentration highlights the substrate’s suitability for quantitative analysis, demonstrating capacity to detect characteristic spectral fingerprints even at trace levels.

The peaks centered around 1,084 and 1,124 cm^−1^ were excluded from the SERS evaluation, as they correspond also to vibrational modes common to cellulose, which appear with low intensity from the SERS substrate. However, the changing ratio of these peaks with violacein concentration suggested a relationship to violacein levels, indicating their potential link to concentration-dependent behavior.

The relative intensities of the bands around 1,084 and 1,124 cm^−1^ display an inversion trend at the lowest tested concentration (10^–7^ M). This is attributed to the decreasing contribution of violacein and the corresponding emergence of cellulose background signals, which become more prominent when the analyte concentration is insufficient. Cellulose typically exhibits a stronger Raman peak at 1,084 cm^−1^, while the 1,124 cm^−1^ band is more representative of violacein. As the analyte concentration increases, the violacein peak at 1,124 cm^−1^ becomes dominant again, confirming its molecular origin and the role of concentration in spectral composition.

**FIGURE 11:**
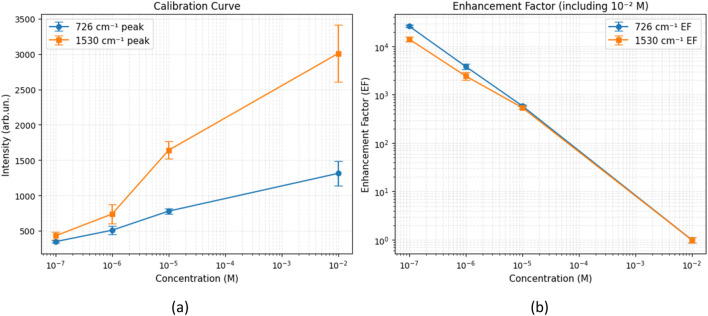
**(a)** Calibration curve of intensity for violacein at 726 cm^−1^ and 1,530 cm^−1^, plotted as a function of concentration (log-log scale). **(b)** Corresponding enhancement factors (EF) computed relative to the normal Raman signal at 10^–2^ M.

This enhancement effect is further supported by the quantitative analysis in Figure 11, which presents (a) the calibration curves based on the integrated SERS intensities and (b) the calculated enhancement factors for the selected violacein bands, both plotted as a function of concentration in the range from 10^–2^ M to 10^–7^ M. We calculated the EF as the average Raman enhancement factor (AEF), which is defined as the ratio between the observed SERS intensity per molecule I_SERS_ and the normal Raman intensity per molecule I_R_:

$$EF=\frac{I_{SERS}/N_{SERS}}{I_{R}/N_{R}}$$

As reported above, a clear normal Raman spectrum cannot be obtained in absence of the Ag film even at the highest violacein concentration of 10^–2^ M (see Figure 9). In this case, in order to still have a EF trend as a function of the concentration we used the spectrum acquired on the 10^–2^ M concentration solution on the Ag coated paper as the I_R_ value, even though this cannot be considered an actual EF. Considering that the same volume of solution at different concentrations was deposited on the substrates and that the measurements were carried out under the same experimental conditions (scattering volume, integration times), the trend of EF depends on the I_SERS_ value assuming that all molecules within the scattering volume contribute to the SERS signal. The intensity values were extracted from three replicate spectra for each concentration, and the error bars represent the standard deviation. The calibration curve covers a concentration range from 10^–2^ M to 10^–7^ M, while at the lowest concentration it is not possible to distinguish signals from the noise, and results obviously 1 at 10^−2^ M. The curve shows a nearly linear relationship on a log-log scale, particularly for the peaks at 1,530 cm^−1^ which shows higher sensitivity. These trends confirm the concentration-dependent SERS response.

The EF values demonstrate a clear increase as concentration decreases. This trend demonstrates a strong amplification, especially at lower concentrations, confirming the efficiency of the AgNP-based substrate in generating electromagnetic hotspots for SERS and the suitability of the system.

As the final observation, we note that the calibration curve for Rhodamine 6G tested with the same device exhibits a similar behavior, with a linear increase in Raman intensity in the log-log scale. However, Rh6G showed higher sensitivity, particularly at low concentrations (10^–10^ M), whereas for violacein on Paper #3, the signal start to become indistinguishable from the noise at 10^−7^ M. This result remains significant when compared to the detectability range reported for other dyes used as probe molecules.

Literature reports limits of Detection (LOD) ranging from 10^–8^ to 10^–5^ M of different dyes, such as 10^–8^ M for Tartrazine (Ai et al., 2018), 10^–7^ M for Sunset Yellow (Ai et al., 2018) and p-aminothiophenol (PATP) (Liu et al., 2016), 10^−6^ M for Thiram (Siebe et al., 2021) and Congo Red (Iancu et al., 2014), 10^–5^ M Cristal Violet (Siebe et al., 2021), as shown in Table 2.

**TABLE 2:** Limit of Detection (LOD) of different dyes used as probe molecule in SERS detection.

Dye	Substrates	Probe molecule, (LOD) M	Ref.
Tartrazine C_16_H_9_N_4_O_9_S_2_Na_3_	flower-shaped Ag NPs in Polyvinylpyrrolidone (PVP)	10^–8^ M	
Sunset yellow C_16_H_10_N_2_Na_2_O_7_S_2_		10^–7^ M	
p-aminothiophenol H_2_NC_6_H_4_SH	Au NPs	10^–7^ M	
Crystal violet C_25_H_30_ClN_3_	AgNPs in hydroxyethyl cellulose (HEC)	10^–5^ M	
Thiram C_6_H_12_N_2_S_4_		10^–6^ M	
Violacein C_20_H_13_N_3_O_3_	AgNPs	10^–7^ M	This work

### 3.4 Computational approaches

SERS spectra of a given molecule can differ substantially from the corresponding Raman spectra. For this reason, the evaluation of the effects of the metal surface on the Raman spectra has been studied by Density Functional Theory (DFT).

**FIGURE 12:**
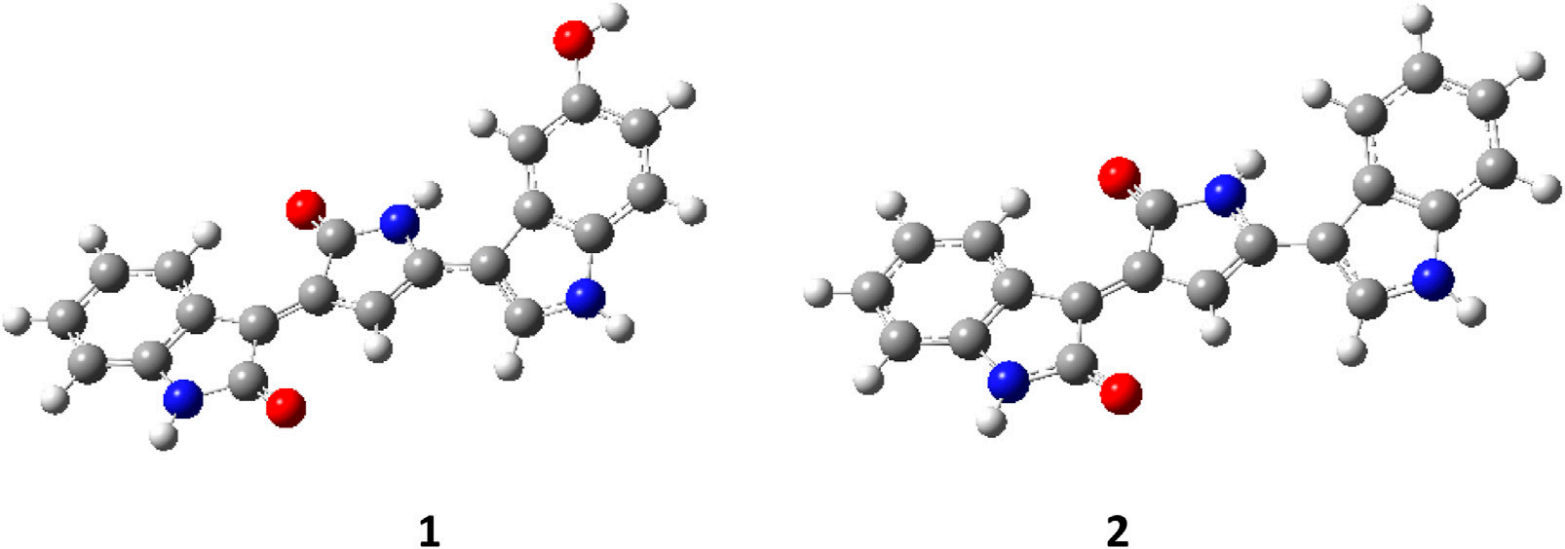
Optimized molecular structure of Violacein **1** and Deoxyviolacein **2**.

DFT calculations were performed with the Gaussian09 (Frisch et al., 2016) software package. We optimized both Violacein (**1**) and Deoxyviolacein (**2**) structures and computed their vibrational spectra at the B3LYP/6-311 (p,d) level of theory in gas phase. Raman spectra were reproduced using Lorentzian line shapes (FWHM = 10 cm^−1^) weighted by the off-resonance Raman activities computed by Gaussian09. The computed wavenumbers were scaled by the empirical factor of 0.983 in the whole range. In Figure 12 are reported the optimized geometries of the **1** (E = −1,160.20276112 Hartree) and **2** (E = −1,084.961941612 Hartree).

The structure of Violacein can be described as composed by a 5-hydroxyindole, oxindole and a 2-pyrrolidone units. In Deoxyiviolacein the 5-hydroxyindole is replaced by an indole unit.

Violacein is a conjugated system so that most of the vibrational modes are expected to be coupled with vibrations involving many units along the whole molecules.

**FIGURE 13:**
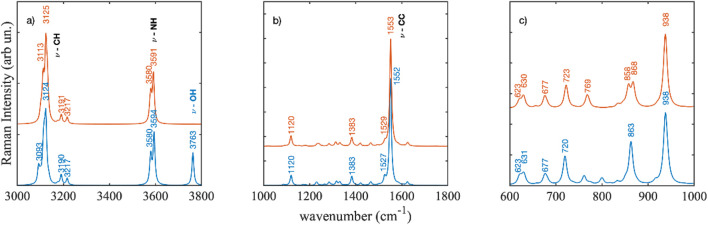
Density functional calculated Raman spectra of violacein structures 1 and 2. Wavenumbers are scaled by a semi-empirical factor of 0.983. Spectral regions are shown from 3000 to 3800 cm^−1^ **(a)**, 1000 to 1800 cm^−1^ **(b)**, and 600 to 1000 cm^−1^ **(c)**.

In Figure 13 are shown the DFT calculated Raman spectra of the two violacein structure **1** and **2**. Comparison is reported in three different spectral regions, panels a), b) and c), owing to the very intense Raman activity of the 1,552 cm^−1^ mode with respect to the remaining Raman modes.

In the high frequency region between 3,000 and 3,800 cm^−1^ (Figure 13a), the calculated spectra showed little differences. Apart from the band at 3,763 cm^−1^ corresponding to OH stretching mode that is missing in the deoxyviolacein calculated spectrum. NH stretching modes appeared between 3,500 and 3,700 cm^−1^ while CH stretching modes were observed between 3,000 and 3,300 cm^−1^. Being such vibrational modes spatially localized major differences were not expected. In the region between 1,000 and 1800 cm^-1^ (Figure 13b) both structures showed the strongest Raman mode at 1,552 cm^−1^ and 1,553 cm^−1^, attributed to the stretching of the two CC bonds connecting the 2-pyrrolidone ring to oxindole and 5-hydroxindole units. In this region the spectra were almost identical the band at 1,383 cm^−1^ was exemplary of a coupled mode assigned to bending mode of CCH and CNH groups present in the oxindole and 2-pyrrolidone units. Similarly, the band at 1,120 cm^−1^ was attributed to the bending mode of CCH and CNH more intense in the 2-pyrrolidone and oxindole unit.

In the low-frequency region, as shown in Figure 13c, some differences were observed. Notably, the mode at 938 cm^-1^ in violacein split into two distinct modes in deoxyviolacein. This splitting occurs due to the activation of an out-of-plane CCH vibration in the indole units, caused by the absence of the hydroxyl group, while all other modes remain unaffected.

**FIGURE 14:**
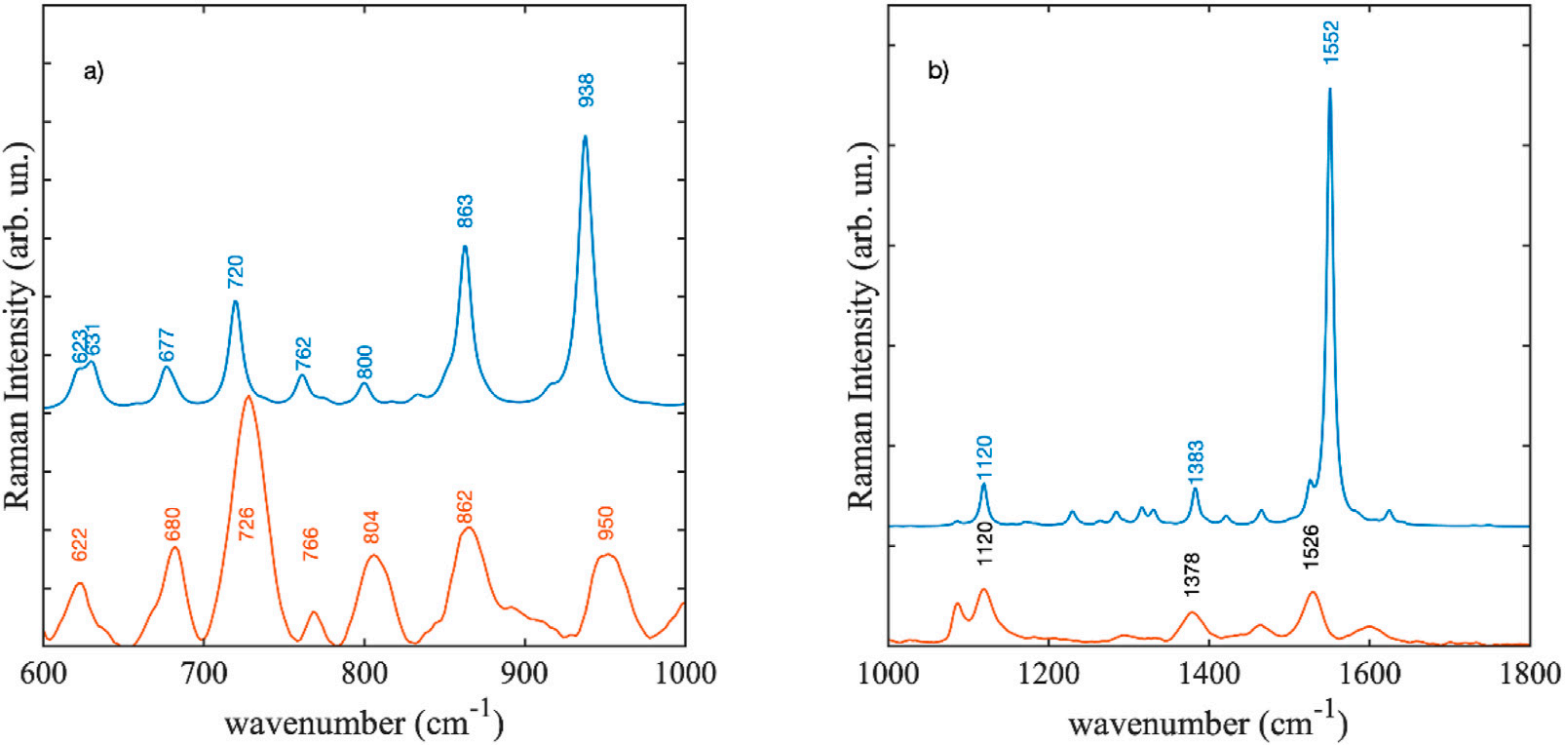
SERS spectrum at 10^−2^ M concentration compared with the DFT calculated Raman spectra Violacein: **(a)** from 600 to 1000 cm^−1^, **(b)** from 1000 to 1800 cm^−1^. The experimental spectrum is scaled to enable easier comparison.

In Figure 14 the experimental SERS spectrum at the concentration level of 10^–2^ M was compared with the calculated DFT Raman spectra of the violacein structures. Two features were readily apparent in the region between 1,000 and 1800 cm^−1^. The most intense peak observed in the experimental spectrum was at 1,526 cm^−1^ this should correspond to the strongest calculated mode of the CC stretching at 1,552 cm^−1^ (Figure 14a). Experimentally it was observed in *Janthinobacterium lividum* extract at 1,552 cm^−1^. In SERS, variations in position and intensity compared to normal Raman or DFT gas-phase calculated spectra are expected, resulting from the interaction of the species with the metallic surfaces, either through covalent bonding or charge transfer mechanisms (Zanchi et al., 2020; Zanchi et al., 2024). Thus, the observed large shift of the CC stretching mode could derive from the establishment of a strong interaction mechanism between violacein and the metal surface. The peak at 1,120 cm^−1^ in the SERS spectrum coincided with a Raman peak of the cellulose substrate onto which the silver films were deposited. However, since this peak increased linearly with the concentration of violacein in the test solutions, we confidently attributed it to the DFT-calculated mode at 1,120 cm^−1^. Additionally, there was a good agreement among the most intense experimental peaks observed at 950, 862, 804 and 726 cm^-1^ that corresponded to the features at 938, 863, and 720 cm^−1^ in the Violacein DFT-calculated spectrum (Figure 14b).

In the context of this work, the presence of a mixture of violacein and deoxyviolacein does not significantly influence the SERS activity on recycled paper coated with silver nanoparticles as the substrate. This characteristic represents a crucial advantage, as the enhanced Raman activity is unaffected by the presence in the mixture of a certain amount of deoxyviolacein, facilitating the application of violacein as an eco-friendly molecular probe.

## 4 Conclusion

This study successfully demonstrated the development of sustainable and flexible SERS-active devices by integrating an innovative deposition method with eco-friendly platforms and probe molecules. The use of recycled paper coated with silver nanoparticles as a SERS substrate proved effective for analytes detection and introduced the potential for material regeneration through a repulping process. This aligns with circular economy principles, minimizing waste, promoting reuse, and meeting the European Commission recycling targets.

The detection of violacein at concentrations as low as 10^–7^ M highlights its strong potential as a sustainable, non-toxic alternative to conventional dyes like Rhodamine 6G. Derived from microbial sources, violacein represents a significant advancement of applying biotechnological techniques and green chemistry principles to spectroscopic analyses. This innovation broadens the scope of SERS applications, including environmental monitoring, diagnostics, and cultural heritage preservation, while reducing the environmental impact associated with traditional probe molecules. Moreover, the portability of the testing instrument enhanced the level of sustainability along with materials and processes involved in this study.

In addition, the combined use of recycled cellulose-based substrates with naturally derived probes obtained through biotechnological techniques marks a significant step toward achieving full sustainability in SERS technology across all components. Ongoing exploration of alternative green solutions, such as silver colloid nanoparticles, further reinforces the integration of green chemistry in spectroscopic analyses. These findings highlight the potential for future advancements in eco-friendly sensor technologies and their application across diverse fields.

## Data Availability

The raw data supporting the conclusions of this article will be made available by the authors, without undue reservation.
